# Elevated and Slowed EEG Oscillations in Patients with Post-Concussive Syndrome and Chronic Pain Following a Motor Vehicle Collision

**DOI:** 10.3390/brainsci11050537

**Published:** 2021-04-24

**Authors:** Derrick Matthew Buchanan, Tomas Ros, Richard Nahas

**Affiliations:** 1 Department of Neuroscience, Carleton University, Ottawa, ON K1S 5B6, Canada; 2 The Seekers Centre, Ottawa, ON K1Z 5Z9, Canada; richard@seekerscentre.com; 3 Laboratory for Neurology and Imaging of Cognition, Department of Neurosciences, University of Geneva, CH1202 Geneva, Switzerland; tomas.ros@unige.ch; 4 Department of Family Medicine, University of Ottawa, Ottawa, ON K1G 5Z3, Canada

**Keywords:** chronic pain, diffuse brain injury, electroencephalography, post-concussive syndrome, support vector machine, EEG, concussion, motor vehicle collision, car accident

## Abstract

(1) Background: Mild traumatic brain injury produces significant changes in neurotransmission including brain oscillations. We investigated potential quantitative electroencephalography biomarkers in 57 patients with post-concussive syndrome and chronic pain following motor vehicle collision, and 54 healthy nearly age- and sex-matched controls. (2) Methods: Electroencephalography processing was completed in MATLAB, statistical modeling in SPSS, and machine learning modeling in Rapid Miner. Group differences were calculated using current-source density estimation, yielding whole-brain topographical distributions of absolute power, relative power and phase-locking functional connectivity. Groups were compared using independent sample Mann–Whitney U tests. Effect sizes and Pearson correlations were also computed. Machine learning analysis leveraged a post hoc supervised learning support vector non-probabilistic binary linear kernel classification to generate predictive models from the derived EEG signatures. (3) Results: Patients displayed significantly elevated and slowed power compared to controls: delta (*p* = 0.000000, *r* = 0.6) and theta power (*p* < 0.0001, *r* = 0.4), and relative delta power (*p* < 0.00001) and decreased relative alpha power (*p* < 0.001). Absolute delta and theta power together yielded the strongest machine learning classification accuracy (87.6%). Changes in absolute power were moderately correlated with duration and persistence of symptoms in the slow wave frequency spectrum (<15 Hz). (4) Conclusions: Distributed increases in slow wave oscillatory power are concurrent with post-concussive syndrome and chronic pain.

## 1. Introduction

Traumatic brain injury (TBI) is a common and important cause of morbidity. Reports indicate over 2.4 million annual United States emergency department visits for TBI in 2013 and 100 thousand in Canada; with slips and falls, followed by motor vehicle accidents (MVA) being the leading causes [1,2,3]. The global estimate of TBI is reported to be between 64 million and 74 million annually [4]. Mild TBI (mTBI), sometimes called concussion, represents about 75% of TBI cases. While most people recover after TBI, it is estimated that about 5.3 million Americans and over 500 thousand Canadians are living with TBI-related disabilities, including many that persist after mTBI—also known as Post-Concussive Syndrome (PCS) [5]. Chronic pain (CP) is an even more prevalent and debilitating condition with a global estimate of up to 20% of adults suffering worldwide, including approximately 25 million Americans and 6 million Canadians [6,7,8]. Nampiaparampil [9] conducted a systemic review of 23 studies involving 4206 patients and found that the comorbidity of CP and mTBI approached 75%, but was only 32% in moderate to severe TBI. Incidence rates of CP following mTBI can be as high as 58% [10]. Recent neuroimaging studies have attempted to uncover the pathophysiology of these conditions independently [11,12,13,14,15,16,17,18,19], but neuroimaging studies in PCS + CP following mTBI are scarce [20,21].

One mechanism of injury in TBI is the rapid change in velocity that generates shear forces which can create lesions in axonal fibers [22,23], a process that has been termed diffuse axonal injury [24]. While diffusion tensor imaging (DTI) is an emerging imaging modality that can identify this characteristic lesion in TBI and PCS [14,16], it is not widely available. Conventional magnetic resonance imaging (MRI) and computed tomography (CT) can identify bleeding and other macro lesions that define moderate and severe TBI, but neither can detect diffuse axonal injury after mTBI [25,26,27]. Quantitative electroencephalography (qEEG) is a relatively less used modality, but its low cost, simplicity, and portability make it well suited for widespread use in the outpatient and community setting. qEEG has been used for decades as an objective measure of abnormalities after brain injury including white matter damage via measurements of electrical changes [15,28,29,30,31]. qEEG also has a long history of use in the evaluation of pain [17,18,19,32,33]. To that end, qEEG will serve our goal of establishing a clinically useful and easily measurable electro-neurophysiological biomarker for concussion and chronic pain. In addition to traditional statistical models, machine learning approaches of biomarker classification in concussion and pain are also becoming increasingly useful [34,35,36,37]. Here, we present evidence both from traditional statistical and machine learning models.

### 1.1. EEG in mTBI/PCS

Brains are constantly fluctuating between states of high (synchronized) and low (desynchronized) local neuronal coupling, which are known to, respectively, generate increases and decreases in EEG spectral power [38]. The dynamic interplay between changes of spectral power within/between different EEG frequencies has been implicated in many cognitive operations, including those of perception, memory, and attention [39,40,41]. Patients with mTBI consistently present symptoms of impaired cognition/attention [20,42,43,44,45,46]. Supporting these behavioral abnormalities, a review paper from Duff [47] demonstrated how mTBI patients express significantly increased magnitude (i.e., power) of slow-wave (delta/theta) EEG across large-scale networks and decreased alpha/beta power. These findings are reinforced by EEG and MRI studies showing positive correlations between increased delta power and white matter lesions plus cognitive dysfunction in PCS [25,28,48,49]. A similar pattern of increased delta power with decreased alpha power has been shown in athletes with sleep disturbances following mTBI [42]. The changes in alpha are further suggested to reflect response inhibition and difficulty with task switching seen in PCS [48,49]. The EEG evidence is again supported by Korn et al. [25], who found, in the normalized power spectra, increased delta and decreased alpha in patients with PCS. Via multimodal imaging, Korn [25] also made some other important observations: single-photon emission computed tomography revealed lesioned blood–brain barrier, and low-resolution brain electromagnetic tomography showed that the lesion site corresponded with abnormal EEG rhythms. In contrast, MRI and CT revealed no differences. Finally, Ponomarev et al. [50] not only observed increases in delta and theta, but also of alpha power in patients with PCS. Given our sample’s PCS, we hypothesized and anticipated to see increases in low-frequency EEG; however, it remains unclear how comorbid CP may additionally interact with this signature.

### 1.2. EEG in Chronic Pain

A detailed systematic review from Pinheiro et al. [51] covered several changes in EEG related to chronic pain. Interestingly, many of these changes are similar to that of PCS. In a sample of neuropathic CP patients vs. controls, Sarnthein et al. [52] report significant increases in absolute delta, theta, alpha, and beta oscillatory power (2–25 Hz). Importantly, the authors included another group of CP patients who were not taking centrally acting medication and found the same increases in power (2–18 Hz), except not in the high beta range (18–25 Hz). Sarnthein [52] also found that these elevations of power and pain attenuated after receiving a therapeutic central lateral thalamotomy. Stern et al. [53] also reported elevated absolute theta and beta power in neurogenic pain, while Vuckovic et al. [54] observed elevated relative theta and alpha power, and absolute alpha power in all brain regions. Additional measures such as decreased peak frequency of theta and alpha rhythms from Bjork et al. [55] also support this EEG phenotype, as it suggests a shift of spectral activity from higher to lower frequency bands. This argument is pursued by Ploner, Sorg and Gross “as slowing of the peak alpha frequency in chronic pain has also been observed, abnormal amplitudes of theta oscillations might basically represent the unspecific slowing of EEG activity” [56]. Consistent changes in theta have been interpreted in the context of the theory of thalamocortical dysrhythmia [57]. This theory proposes that thalamic theta oscillations influence cortical inhibition, resulting in increased gamma power associated with positive neurological symptoms such as pain [55]. That said, it is still unclear to what extent other frequency bands (i.e., beta and gamma) are affected. The common feature, however, is the elevation of absolute EEG power in patients with pain versus healthy controls. This is also supported using other neuroimaging modalities such as magnetoencephalography (MEG). Lim et al.’s [18] use of MEG was consistent, revealing elevated absolute theta, beta, and gamma power, though concurrently decreased alpha. Overall, a general trend for CP appears to be increased broadband power.

### 1.3. Hypotheses for PCS and CP

Overall, it seems PCS most prominently presents with elevated low frequency activity of the delta and theta bands. CP seems to generally elevate broadband power with alpha being a frequent exception. As a result, we hypothesized a “combined” effect of increased slow and fast (i.e., broadband) EEG power. Due to the nature of mTBIs resulting from car accidents, such as the shear force mechanisms, diffuse axonal injury, and the orientation of the patient upon collision, it is also likely that the changes in EEG power will be diffuse. Furthermore, we sought to answer, for the first time, whether changes in EEG power might be reflected by functional connectivity in this comorbid sample. While there are a few reports measuring functional connectivity using phase-locking values and other measures such as MEG for PCS and CP separately, their results are so far inconsistent [11,58,59].

## 2. Materials and Methods

### 2.1. Participants

We conducted EEG assessments on fifty-seven patients (mean age 44.6, SD 11.2; 36 females) who were referred to The Seekers Centre, a community-based pain management clinic in Ottawa, Canada. Some patient information is summarized below in Table 1. Inclusion criteria for this study were: patients must have a physician diagnosis of CP and PCS with onset beginning post-MVA, and patients must have sustained mTBI due to MVA. Exclusion criteria for this study were: patients with other causes of pain, other causes of brain injury, or patients with identifiable neurological illnesses. All patients included in this study were referred to the clinic for Chronic Pain and PCS persisting at least three months following an mTBI caused by a motor vehicle collision. Similarly, the morbidity of each patient at the time of their EEG assessment included CP + PCS, and occasionally, a comorbidity such as PTSD, anxiety, or depression. Fifty-six percent of our patients had at least one of these comorbidities. However, a statistical analysis following the same methodology as described below in Section 2.4 revealed that there were no significant differences in EEG signals between patients with CP + PCS (*n* = 25) versus those with CP + PCS + comorbidity (*n* = 32). In the same way, there were no significant differences in EEG signals between patients who lost consciousness (*n* = 30) and those who retained consciousness (*n* = 25).

Fifty-four nearly age- and sex-matched controls (mean age of 43.5. SD 9.4; 38 females) from the Human Brain Institute (HBI) normative database were used for comparison [60]. The control group consisted of healthy people with no history of mTBI or any other physiological, neurological, or neuropsychiatric condition. The EEG acquisition and analysis were the same for controls and patients. The HBI control data were acquired before the start of our study. However, the methods and equipment we used for the patient sample were identical to the methods and equipment used for the controls from the HBI database.

This study was approved by the Bruyère Research Ethics Board. All participants provided written informed consent (ethics approval code: # M16-14-009).

### 2.2. EEG Data Acquisition

EEG data were recorded with a 19-channel EEG Quik-Cap (Electrocap International Inc., Eaton, OH, USA) using Ag/AgCl surface electrodes connected to a Mitsar 21-channel EEG amplifier (Mitsar-201, CE0537, Mitsar, Ltd., St Petersburg, Russia) running on WinEEG software according to the 10–20 international system [61]. The ground electrode was placed on the right ear and reference electrode on the left. Electrogel was applied to each electrode via syringe with impedance maintained below 5 kΩ. EEG data were recorded and digitized at a sampling frequency of 250 Hz. As noted above in Section 2.1, the same protocol was followed for patients and controls.

One day prior to the assessment, participants were telephoned and told to avoid caffeine and tobacco, to get normal sleep, and to take their medications as usual. For our patient population, this mostly included anti-inflammatory pain medications, occasionally opiate-based pain medications, in some cases of comorbidity antidepressants or antipsychotics, and rarely anticonvulsants. Assessments were performed with patients seated upright in a dimly lit, unshielded room with an EEG technician present. Verbal instructions included sitting upright, remaining still, avoiding jaw clenching and blinking, fixating gaze and remaining awake. Resting-state qEEG recordings were made with eyes open for a duration of 3 min. Pauses (<60 s each) allowed patients to move or blink as needed.

### 2.3. EEG Data Analysis

Eye blinks and other stereotypical artifacts were removed by independent component analysis (ICA) via EEGLAB and the Infomax algorithm (blinking and lateral eye movements) [62,63]. Statistically defined artifact rejection was then carried out with the FASTER method, removing segments based on extreme deviations of amplitude and variance from the mean [64].

After carefully removing artifacts in both control and patient recordings, the data were analyzed using Neurophysiological Biomarker Toolbox (NBT) and MATLAB 2018b software [65,66]. First, the EEG signal was re-referenced to current-source density (CSD) [67]. Doing this reduces the amount of volume conduction in the signal. The EEGs were then band-pass filtered between 1 and 45 Hz, and Welch’s method was used to calculate the power spectral density (PSD) of six frequency bands: 1–4 Hz (delta), 4–7 Hz (theta), 7–13 Hz (alpha), 13–15 Hz (low beta), 15–30 Hz (high beta), and 30–45 Hz (gamma). Values for absolute power, relative power, and phase-locking were calculated for each frequency band at each electrode. From these values, whole-brain averages across the 19 electrodes were computed for patients and controls. Given that the location of injury on the head is heterogeneous (i.e., front end collision, rear end collision, side impact), whole-brain averages/medians were used for statistical analyses.

### 2.4. Statistical Analysis

**Figure 1:**
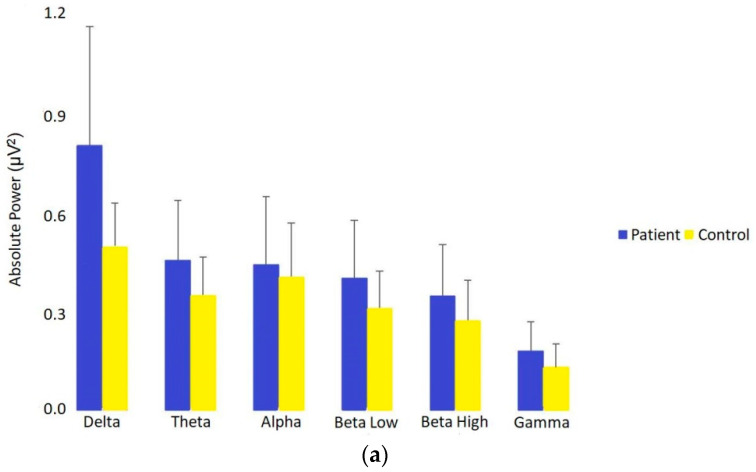

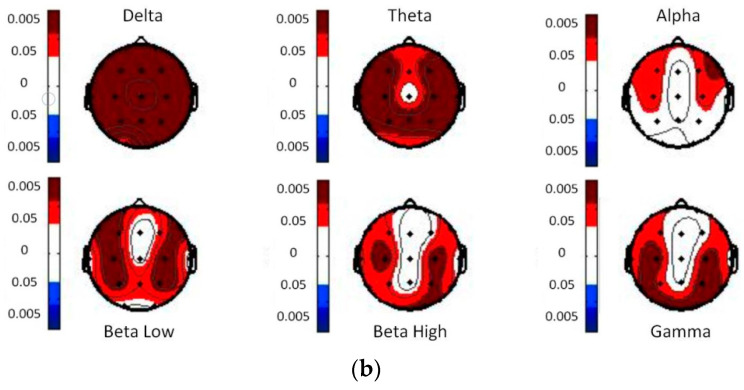
(**a**) Absolute power differences (expressed as medians) across all EEG frequency bands between patients with PCS + CP versus healthy age- and sex-matched controls. Absolute power was significantly elevated in patients across all frequency bands (Bonferroni-corrected alpha: 0.05/6 = 0.0083), except for alpha. Delta waves were the most affected. (**b**) Absolute power differences across all frequency bands between patients with PCS + CP versus healthy age- and sex-matched controls using independent sample, two-tailed Mann–Whitney U test comparisons and color-coded uncorrected *p*-values overlaid on EEG headmaps. Dark red indicates increases between patients versus controls at <0.005, dark blue the converse, and white means no significant change. All frequency bands are significantly increased.

**Figure 2:**
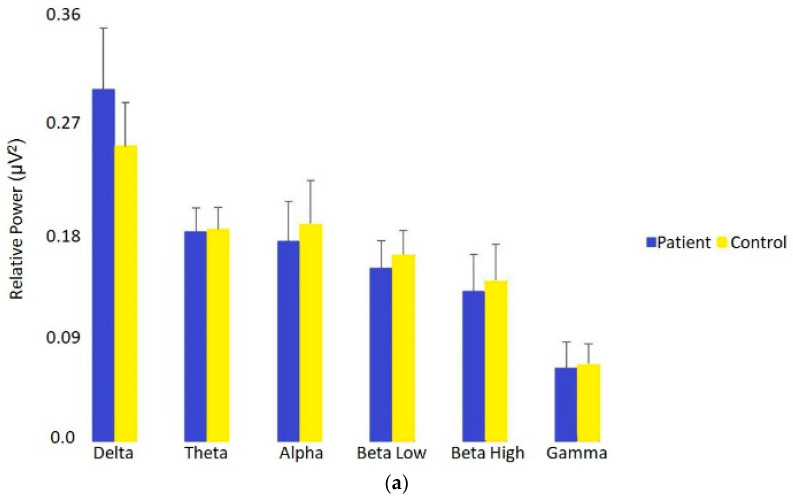

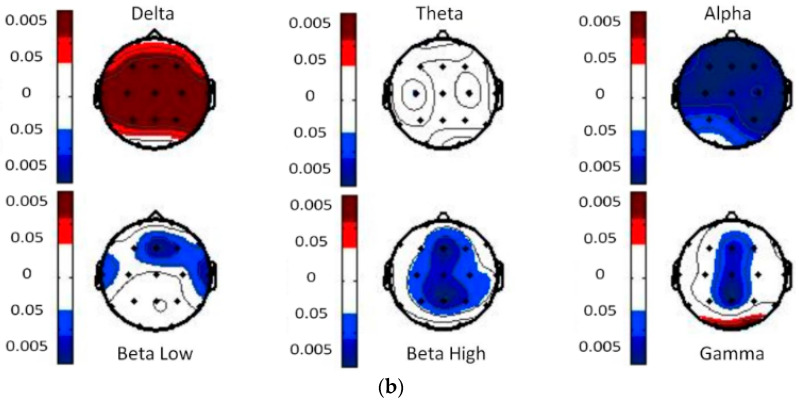
(**a**) Relative power differences (expressed as medians) across all EEG frequency bands between patients with PCS + CP versus healthy age- and sex-matched controls. Relative delta power was significantly elevated in patients, whereas alpha was significantly decreased (Bonferroni-corrected alpha: 0.05/6 = 0.0083). (**b**) Relative power differences across all frequency bands between patients with PCS + CP versus healthy age- and sex-matched controls using independent sample, two-tailed Mann–Whitney U test comparisons and color-coded uncorrected *p*-values overlaid on EEG headmaps. Dark red indicates increases between patients versus controls at <0.005, dark blue the converse, and white means no significant change. Delta is significantly elevated, and alpha is significantly decreased.

Statistical analyses were performed using SPSS Statistics Version 20.0 [68]. Kolmogorov–Smirnov tests were conducted to test for normality. Since normality was violated for the majority of the variables, medians were used and nonparametric two-tailed Mann–Whitney U tests (Bonferroni corrected alpha: 0.05/6 = 0.0083) were performed to identify differences in absolute power, relative power, and phase-locking between patients and controls for each frequency band (Table 2; Figure 1a and Figure 2a). Additionally, effect sizes (*r*) were calculated based on the Z statistic where *r* is the magnitude of the difference between groups. Uncorrected *P*-values below 0.05 and 0.01 were anatomically clustered as increases (red) and decreases (blue) on topographic plots (Figure 1b and Figure 2b). Lastly, Pearson correlations were calculated based on the duration of the patients’ diagnoses and their absolute EEG power (Table 3).

### 2.5. Support Vector Analysis

Finally, a post hoc supervised learning support vector non-probabilistic binary linear kernel classification analysis was conducted using an open-sourced Java-based software called Rapid Miner Studio 9.5 [69]. The goal was to test the discriminatory power of various EEG signals in predicting patients versus controls within our cohort using a support vector approach. Based on the results of our statistical analysis, we specifically created models containing the EEG signals that had the most significant differences between groups: absolute power, absolute delta power, and absolute delta and theta power. Each model consisted of at least 111 rows and 21 columns (i.e., absolute delta power at each of the 19 electrode sites, plus the whole-brain average across all electrodes, plus the binary classifier patient or control, for all 111 participants). Each model tested the discriminatory power of the included biomarkers on predicting the binary class patient or control.

Based on our initial analysis, three support vector models were created: absolute power values for six discrete frequency bands (111 × 21 × 6); absolute power for delta and theta frequency bands (111 × 21 × 2); and absolute power in the delta frequency band (111 × 21).

The following process was applied to each of the three above models. The process began with the creation of a training and validation set, where the validation set was used in a multiple hold-out performance calculation. The total dataset was split 60:40 into a training and validation set using automatic stratified sampling. For our sample, this was equal to 66 in the training set and 45 in the validation set. Then, the training set was trained using cost-sensitive scoring whereby a 10-fold multiplier generated 10 artificial datapoints for each real datapoint that was being predicted. The distributions of the artificial datapoints were then used to define the confidence in terms of their proximity to the real value being predicted. These confidences were then averaged and used to derive the expected cost. Values with the lowest costs were used to define the ultimate prediction within the training set. The values closest to the optimally separating hyperplane are considered the optimal feature set. For the validation set, the known trained values were applied to the unlabeled hold out set. Then, the optimal feature set was applied to the training data and the validation model before applying the final prediction model. Lastly, the final prediction model was derived by once again applying the aforementioned 10-fold cost-sensitive scoring method to the validation set based on the optimal training set.

## 3. Results

### 3.1. Statistical Analysis

#### 3.1.1. Absolute Power in Patients vs. Controls

The most obvious and consistent difference between patients and controls was seen in absolute power (Figure 1a,b). Absolute power is a measure of the electrical output of synchronous neuronal firing relative to a reference. Independent two-tailed Mann–Whitney U tests revealed that patients’ EEG power relative to controls was significantly elevated across delta (U = 2606, *p* = 0.000000, *r* = 0.6), theta (U = 2246, *p* = 0.00003, *r* = 0.4), beta low (U = 2055, *p* = 0.002, *r* = 0.29), beta high (U = 2007, *p* = 0.006, *r* = 0.26), and gamma (U = 2049, *p* = 0.003, *r* = 0.29), but not quite for alpha (U = 1910, *p* = 0.029, *r* = 0.21) with a corrected significance threshold of 0.0083. Of note are the high and medium effect sizes for delta and theta, respectively. This preponderance of hypersynchronous and low-frequency EEG is further summarized in Table 2. Figure 1a also shows the marked power increase in patients vs. controls, and regional differences based on electrode location can be seen topographically in Figure 1b. The color scale of each brain topogram is such that smaller *p*-values equal a darker red (increase) or darker blue (decrease) based on standard deviation units relative to the distribution of expected normal values.

#### 3.1.2. Relative Power

Relative power is a ratio metric of absolute power which measures the ratio power at one frequency relative to the total power of all other frequencies. As shown in Figure 2a,b, relative power in participants with PCS and CP vs. healthy controls was again increased in the delta band (U = 2304, *p* = 0.000006, *r* = 0.43) but now decreased in the alpha band (U = 2129, *p* = 0.0005, *r* = 0.33) with medium effect sizes. There were no significant differences in relative power for any of the other frequency bands, though small effects were present in beta low and beta high.

#### 3.1.3. Phase-Locking Connectivity

Phase-locking is a measure of functional connectivity that is derived by comparing electrical activity in a single channel in relation to other channels at a specific moment in time. Independent two-tailed Mann–Whitney U tests revealed patients’ phase-locking did not significantly differ from controls in any frequency band but there were non-significant trends towards decreased connectivity in the alpha band (U = 1874, *p* = 0.048, *r* = 0.19) and increased connectivity in delta (U = 1838, *p* = 0.078, *r* = 0.17). Further results are summarized in Table 2.

#### 3.1.4. Correlation between Absolute Power and Duration of Symptoms

The Pearson correlations summarized in Table 3 suggest a weak to moderate positive correlation of elevated power and duration of symptoms. The correlation was strongest in the delta band (r = 0.35, *p* < 0.01). Theta (*r* = 0.31, *p* < 0.05), alpha (*r* = 0.32, *p* < 0.05), and low beta (*r* = 0.32, *p* < 0.05) were also significantly correlated but high beta and gamma were not.

### 3.2. Support Vector Machines

Support vector machine learning most successfully classified participants using a combination of delta and theta absolute power: accuracy 87.6% (SD = 7), AUC 90% (SD = 14.9), precision 100% (SD = 0), F measure 85.1% (SD = 8.7), sensitivity 75% (SD = 14.4), specificity 100% (SD = 0). SVM results were also robust across other biomarkers including just delta absolute power: accuracy 81.9% (SD = 6), AUC 86.1% (SD = 12.7), precision 100% (SD = 0), F measure 78.5% (SD = 7.1), sensitivity 65% (SD = 9.1), specificity 100% (SD = 0); and absolute power using all frequency bands: accuracy 78.6% (SD = 6.5), AUC 92.8% (SD = 7.2), precision 90% (SD = 13.7), F measure 77.5% (SD = 7.1), sensitivity 71.7% (SD = 18.3), specificity 86.7% (SD = 18.3).

## 4. Discussion

The present study identified diffuse elevated broadband power, especially in the low-frequency spectrum, as a primary feature of patients with post-concussive syndrome and chronic pain. Statistical and machine learning analysis supported our a priori hypothesis that PCS combined with CP would lead to a compounding increase in absolute power in low (PCS) and high (CP) frequency bands. Diffuse delta (*r* = 0.6) and theta (*r* = 0.4) power were the most prominent significant differences between patients and controls, and each boasted relatively large effect sizes; and our support vector model using delta and theta power was able to discriminate patients from controls with up to 87.6% accuracy. Although it is still not clinically viable, this level of SVM classification is superior to the current literature for mTBI [34,35] and on par for pain [37]. This is also the first demonstration of a potential biomarker for comorbid PCS + CP from resting state EEG.

This important first account of the combined abnormalities of PCS + CP on electrophysiological signatures provides rationale for further investigation of delta and theta power as potential clinically useful biomarkers. In our future research, we aim to validate these candidate markers in a population of acute concussion and pain to see if they can predict the development of PCS + CP and subsequently predict treatment response. Additionally, this will require us to extend our results by validating the aforementioned signatures at the individual patient level. For our results to be fully translatable into clinical practice, it will be important to extend our group level findings to the single subject level. In the present experiment, the interpretation of our results is limited to the group level. In other words, a group of PCS + CP vs. healthy controls would be expected to show the patterns found in this paper (i.e., high delta, high theta, etc.), but a single subject may not always fit this pattern.

Although there is still a great deal of research required to validate these signatures as viable biomarkers of PCS + CP, our present study made some important contributions and improvements on the previous literature. Firstly, our study was conducted in a relatively large, nearly age- and sex-matched sample with a homogeneous mechanism of injury. A homogenous mechanism of injury is important because of the biomechanical and psychosocial factors that distinguish MVA injury from other causes of PCS [23,70,71]. As such, homogenous samples may provide additional value to research data obtained from brain injury patients. That being said, there is still a heterogeneous nature of mTBIs caused by car accidents as the orientation of the impact can vary. This is why it is important to consider the global diffusivity of the elevated EEG power presented in our results. Secondly, we demonstrated significant weak to moderate positive correlations between globally distributed absolute power and duration of symptoms across lower frequency bands (1–15 Hz) which suggests progression of disease, as pain and suffering continue to affect the brain over months and years. This correlation seems to represent an ongoing change in the brain that occurs throughout the duration of the illness. This supports the hypothesis that whole-brain absolute power of 1–15 Hz oscillations may be useful in measuring response to treatment. Thirdly, since the first report of EEG changes after concussion from Walker et al. [72], there have been remarkable advancements in the algorithms that are used to detect abnormalities. Nonetheless, the clinical utility of EEG findings in TBI have been limited by: inconsistent findings reported by preliminary studies, differences in methodology between studies, and complex parameters that have been proposed as clinical biomarkers. The present study overcame each of these limitations by offering a methodology that is easily replicable and a biomarker that is easy to produce and understand. Consequently, with further validation, it would not be difficult for translation into concussion screening or into outpatient treatment centers. Finally, this study was conducted in real-world clinical practice where strict avoidance of medication is seldom practical as limiting its use may also create withdrawal with a concomitant influence on EEG data. Thus, our study adopted the approach of many previous neuroimaging studies, such that we instructed patients to continue their medication as usual (occasionally including anti-inflammatory pain medications, opiate-based pain medications, and in some cases of comorbidity, antidepressants or antipsychotics, and rarely anticonvulsants). While it is possible that some part of the between-group difference in global absolute power is due to the impact of medication on brain physiology, conversely, they may in fact mitigate this difference by providing symptom reduction. This real-world component of our study lends to its ecological and thus translational validity. Additionally, Sarnthein et al. [52] provide evidence that increased power was consistent in groups with or without centrally acting pain medication. Therefore, the same should be expected from our cohort at the group level. It is also expected, however, that there will of course be some differences in EEG at the individual level (perhaps explained by medications or comorbidities within the patient group), but at the group level, the variance between patients’ and controls’ EEGs in our sample seem to be best explained by their chronic pain and their post-concussive syndrome.

Delta is understood as an unconscious rhythm largely generated by the brainstem and characterized by drowsiness and sleep-like states. When seen in brain-injured patients, it may represent lesions and/or brain inflammation [28,73]. The significant and generalized nature of the increased delta synchronization reported in this study provides further evidence that aberrant delta activity may be an indicator of diffuse axonal injury. Significant slowing of patient EEG relative delta power (*r* = 0.43) and a decrease in alpha (*r* = 0.33) may be indicative of a compensatory shift in the brain toward slower oscillations as there are increased energy requirements for maintaining neuronal oscillations in the high-frequency bands [74]. Therefore, our results may be representative of the brain’s attempt to conserve neuronal energy in an already injured system. More generally, the broadband increase in absolute power likely suggests abnormally elevated synchronization of intracortical neuronal activity [38]. This finding, combined with the trend toward decreased alpha phase-locking, may indicate that as shorter-range connectivity/synchronization (i.e., EEG power) increases, longer-range connectivity (i.e., phase-locking) decreases. Further research will have to elucidate this hypothesis.

## 5. Conclusions

Quantitative EEG is a low-cost, non-invasive measure useful for characterizing PCS and CP. Using delta and theta power, we successfully differentiated patients from controls with high accuracy, improving on the current literature. Increased delta and theta power may be clinically relevant diagnostic indicators. These markers may even have prognostic value, and could be combined with baseline and post-injury measures in order to assess treatment response and guide treatment strategies, including medication, brain stimulation, neurofeedback, or other rehabilitative interventions [75,76].

## Figures and Tables

**Table 1:** Patients’ MVA and morbidity.

Patient	Age	Sex	Direction of Impact	LOC	Location in Vehicle	Diagnosis
1	38	M	Front	No	Driver	CP, PCS, PTSD
2	52	F	Rear	No	Front passenger	CP, PCS, anxiety
3	51	M	Rear	No	Driver	CP, PCS, depression
4	46	M	Side	Yes	Driver	CP, PCS, PTSD
5	60	F	Front	Yes	Driver	CP, PCS, PTSD
6	53	F	Side	No	Driver	CP, PCS, PTSD
7	43	F	Vehicle rollover	No	Driver	CP, PCS
8	42	F	Rear	No	Front passenger	CP, PCS, PTSD
9	23	F	Side	Yes	Driver	CP, PCS, anxiety
10	44	M	Rear	No	Driver	CP, PCS
11	55	F	Pedestrian hit by car	Yes	Pedestrian (hit by car, walking)	CP, PCS, PTSD
12	56	F	Side	No	Driver	CP, PCS
13	57	F	Rear	No	Front passenger	CP, PCS
14	48	F	Rear	No	Driver	CP, PCS
15	40	M	Rear	No	Driver	CP, PCS, depression, PTSD
16	48	F	Rear	Yes	Driver	CP, PCS
17	24	M	Rear	No	Driver	CP, PCS
18	52	M	Pedestrian hit by car	Yes	Pedestrian (hit by car, walking)	CP, PCS, depression
19	65	M	Side	Yes	Driver	CP, PCS
20	54	F	Rear	No	Driver	CP, PCS, anxiety
21	32	M	Rear	Yes	Driver	CP, PCS
22	24	F	Rear ended then hit another car head on	Yes	Driver	CP, PCS, depression, anxiety
23	21	F	Rear	No	Driver	CP, PCS
24	45	M	Side	No	Front passenger	CP, PCS, anxiety
25	52	M	Rear	No	Driver	CP, PCS
26	40	F	Rear	Yes	Driver	CP, PCS, PTSD
27	34	F	Rear ended another vehicle	Yes	Front passenger	CP, PCS, anxiety
28	55	F	Side	Yes	Driver	CP, PCS
29	58	F	Side	No	Driver	CP, PCS, anxiety
30	37	M	Side	Yes	Driver	CP, PCS, PTSD, depression, anxiety
31	45	F	on bus	Yes	Front passenger	CP, PCS
32	23	F	Front	Yes	Driver	CP, PCS
33	43	F	Rear	No	Driver	CP, PCS
34	40	M	Side	Yes	Front passenger	CP, PCS, depression
35	45	F	Rear	Yes	Driver	CP, PCS
36	48	M	Front	No	Driver	CP, PCS
37	52	M	Front	No	Front passenger	CP, PCS, PTSD
38	36	F	Side	Yes	Driver	CP, PCS
39	32	F	Front	No	Driver	CP, PCS, PTSD
40	45	M	Side	No	Driver	CP, PCS, PTSD
41	40	F	Head on	No	Driver	CP, PCS, PTSD
42	45	F	Side	No	Moose hit driver	CP, PCS, anxiety
43	12	M	Side	No	Front passenger	CP, PCS, anxiety
44	61	F	Side	Yes	Driver	CP, PCS, PTSD
45	49	M	Front	NA	Driver	CP, PCS, PTSD
46	49	F	Front	Yes	Driver	CP, PCS
47	48	F	Rear	NA	Driver	CP, PCS
48	52	M	Front	Yes	Rear passenger	CP, PCS, anxiety
49	52	F	Rear	Yes	Driver	CP, PCS, PTSD
50	53	M	Side	Yes	Driver	CP, PCS
51	38	F	Front	Yes	Front passenger	CP, PCS, PTSD
52	31	F	Rear	Yes	Front passenger	CP, PCS, anxiety
53	51	F	Pedestrian hit by car	Yes	Pedestrian (hit by car, jogging)	CP, PCS
54	56	F	Rear	Yes	Driver	CP, PCS
55	60	F	Rear	Yes	Driver	CP, PCS
56	45	M	Side	Yes	Bicyclist (hit by car)	CP, PCS, anxiety
57	42	F	Rear	Yes	Driver	CP, PCS, PTSD

Information regarding patient’s motor vehicle accident and morbidity. LOC = loss of consciousness, CP = chronic pain, PCS = post-concussive syndrome, PTSD = post-traumatic stress disorder, NA = not available.

**Table 2:** qEEG signature comparisons between patients and controls.

	qEEG Parameters								
	Absolute Power µv^2^			Relative Power			Phase-Locking		
Frequency Bands	Patient Mdn (IQR) 25th 75th	Control Mdn (IQR) 25th 75th	*p*-value (*r*)	Patient Mdn (IQR) 25th 75th	Control Mdn (IQR) 25th 75th	*p*-value (*r*)	Patient Mdn (IQR) 25th 75th	Control Mdn (IQR) 25th 75th	*p*-value (*r*)
Delta (1–4 Hz)	0.79 (0.58) (1.03)	0.49 (0.44) (0.59)	**0.000000** 0.6	0.30 (0.26) (0.34)	0.25 (0.23) (0.28)	**0.000006** 0.43	0.28 (0.28) (0.28)	0.28 (0.28) (0.28)	0.078 0.17
Theta (4–7 Hz)	0.45 (0.35) (0.59)	0.35 (0.29) (0.39)	**0.00003** 0.4	0.18 (0.16) (0.19)	0.18 (0.16) (0.19)	0.75 0.03	0.25 (0.25) (0.25)	0.25 (0.25) (0.25)	0.42 0.07
Alpha (7–13 Hz)	0.44 (0.36) (0.55)	0.4 (0.28) (0.52)	0.029 0.21	0.17 (0.15) (0.19)	0.19 (0.17) (0.23)	**0.0005** 0.33	0.19 (0.19) (0.20)	0.207 (0.19) (0.21)	0.048 0.19
Low Beta (13–15 Hz)	0.40 (0.33) (0.48)	0.31 (0.25) (0.43)	**0.002** 0.29	0.15 (0.13) (0.16)	0.16 (0.14) (0.17)	0.067 0.17	0.15 (0.15) (0.16)	0.15 (0.15) (0.16)	0.74 0.03
High Beta (15–30 Hz)	0.34 (0.27) (0.45)	0.27 (0.22) (0.37)	**0.006** 0.26	0.13 (0.11) (0.15)	0.14 (0.12) (0.15)	0.077 0.17	0.13 (0.13) (0.13)	0.13 (0.13) (0.14)	0.10 0.15
Gamma (30–45 Hz)	0.18 (0.13) (0.24)	0.13 (0.09) (0.59)	**0.003** 0.29	0.06 (0.05) (0.8)	0.07 (0.05) (0.8)	0.77 0.03	0.10 (0.10) (0.11)	0.10 (0.10) (0.11)	0.69 0.04

Group medians of 19-electrode channel global average. Whole-brain qEEG differences across all frequency bands between patients with PCS + CP versus healthy age- and sex-matched controls using independent sample, two-tailed Mann–Whitney U test comparisons. Exact *p*-values are reported. The Bonferroni correction was applied for each frequency band yielding a 0.05/6 = 0.0083 significance threshold. Therefore, *p* < 0.0083 is significant. Significant *p* values are marked in bold font. Effect sizes *r* are reported. Mdn = median, IQR = interquartile range.

**Table 3:** Correlation between absolute power and duration of symptoms.

Absolute Power Frequency Bands	Pearson Correlation	*p*-Value
Delta (1–4 Hz)	0.35	*p* < 0.01
Theta (4–7 Hz)	0.31	*p* < 0.05
Alpha (7–13 Hz)	0.32	*p* < 0.05
Low Beta (13–15 Hz)	0.32	*p* < 0.05
High Beta (15–30 Hz)	0.06	n.s
Gamma (30–45 Hz)	−0.06	n.s

Correlations between absolute power and duration of symptoms (time since MVA) in patients with PCS + CP.

## Data Availability

The data for this study are privately owned and therefore, not publicly available. Data may be requested privately via communication with the corresponding author.
